# Participants’ Engagement and Satisfaction With a Smartphone App Intended to Support Healthy Weight Gain, Diet, and Physical Activity During Pregnancy: Qualitative Study Within the HealthyMoms Trial

**DOI:** 10.2196/26159

**Published:** 2021-03-05

**Authors:** Johanna Sandborg, Pontus Henriksson, Erica Larsen, Anna-Karin Lindqvist, Stina Rutberg, Emmie Söderström, Ralph Maddison, Marie Löf

**Affiliations:** 1 Department of Biosciences and Nutrition Karolinska Institutet Huddinge Sweden; 2 Department of Health, Medicine and Caring Sciences Linköping University Linköping Sweden; 3 Division of Health, Medicine and Rehabilitation Department of Health, Education and Technology Luleå University of Technology Luleå Sweden; 4 Institute for Physical Activity and Nutrition School of Exercise and Nutrition Sciences Deakin University Burwood, Melbourne Australia

**Keywords:** pregnancy, gestational weight gain, mHealth, telemedicine, digital health, mobile health, eHealth, smartphone intervention, mobile application, smartphone application, engagement, physical activity, exercise, nutrition, diet, qualitative, thematic analysis

## Abstract

**Background:**

Excessive gestational weight gain (GWG) is common and associated with negative health outcomes for both mother and child. Mobile health–delivered lifestyle interventions offer the potential to mitigate excessive GWG. The effectiveness of a smartphone app (HealthyMoms) was recently evaluated in a randomized controlled trial. To explore the users’ experiences of using the app, a qualitative study within the HealthyMoms trial was performed.

**Objective:**

This qualitative study explored participants’ engagement and satisfaction with the 6-month usage of the HealthyMoms app.

**Methods:**

A total of 19 women (mean age: 31.7, SD 4.4 years; mean BMI: 24.6, SD 3.4 kg/m^2^; university degree attainment: 13/19, 68%; primiparous: 11/19, 58%) who received the HealthyMoms app in a randomized controlled trial completed semistructured exit interviews. The interviews were audiorecorded and fully transcribed, coded, and analyzed using thematic analysis with an inductive approach.

**Results:**

Thematic analysis revealed a main theme and 2 subthemes. The main theme, “One could suit many: a multifunctional tool to strengthen women’s health during pregnancy,” and the 2 subthemes, “Factors within and beyond the app influence app engagement” and “Trust, knowledge, and awareness: aspects that can motivate healthy habits,” illustrated that a trustworthy and appreciated health and pregnancy app that is easy to use can inspire a healthy lifestyle during pregnancy. The first subtheme discussed how factors within the app (eg, regular updates and feedback) were perceived to motivate both healthy habits and app engagement. Additionally, factors beyond the app were described to both motivate (eg, interest, motivation, and curiosity) and limit (eg, pregnancy-related complications, lack of time) app engagement. The second subtheme reflected important aspects, such as high trustworthiness of the app, increased knowledge, and awareness from using the app, which motivated participants to improve or maintain healthy habits during pregnancy.

**Conclusions:**

The HealthyMoms app was considered a valuable and trustworthy tool to mitigate excessive GWG, with useful features and relevant information to initiate and maintain healthy habits during pregnancy.

**Trial Registration:**

ClinicalTrials.gov NCT03298555; https://clinicaltrials.gov/ct2/show/NCT03298555

**International Registered Report Identifier (IRRID):**

RR2-10.2196/13011

## Introduction

### Excessive Gestational Weight Gain

Excessive gestational weight gain (GWG) is associated with an increased risk of several complications in both mother and child [1,2]. In the short term, these include gestational hypertension, preeclampsia, gestational diabetes mellitus, cesarean delivery, infant macrosomia, preterm birth, and neonatal morbidity and mortality [3,4]. Excessive GWG is also associated with postpartum weight retention and an increased risk of obesity in the offspring, which are indicators of long-term complications [2,4]. Currently, approximately 50% of pregnant women exceed the commonly applied recommendations from the National Academy of Medicine [5] and thereby have an increased risk of different pregnancy-related complications [4,6].

### Mobile Health Interventions to Prevent Excessive GWG

While there is strong evidence that a healthy diet, physical activity, or both during pregnancy can reduce the risk of excessive GWG [7,8], conventional in-person programs are time consuming and costly to deliver and have limited reach. Behavior change interventions delivered through mobile phones address some of these issues, as they are easy to access, require fewer resources to deliver once developed, and have greater reach [9-11]. Previous studies have shown positive effects of mobile health (mHealth) programs on weight loss in adults [12], and several pilot studies have shown that mHealth interventions also have the potential to reduce excessive GWG in pregnant women [13-15].

In addition to quantitative evaluations of the effectiveness of mHealth programs on GWG and health behaviors, it is essential to examine user engagement and satisfaction with mHealth programs to facilitate future development, tailoring, and improvements. However, to the best of our knowledge, only 1 study has examined user engagement and satisfaction of an mHealth program for supporting healthy GWG [16]. In brief, in this qualitative pilot study [16], participants (n=13) tested an app (SmartMoms Canada) for 2 to 4 weeks with the aim of evaluating the app prior to testing it in a multicenter study. No previous studies have examined engagement and satisfaction related to long-term usage (ie, 6 months) of an mHealth program to support a healthy weight gain and lifestyle during pregnancy. We have recently developed [17] and evaluated the effectiveness [18] of the smartphone app HealthyMoms, which sought to promote healthy weight gain, diet, and physical activity in pregnancy during 6 months in a randomized controlled trial. This paper reports the findings of a qualitative study that explored the engagement and satisfaction with the HealthyMoms app within the trial.

## Methods

### Study Design and Recruitment

**Figure 1:**
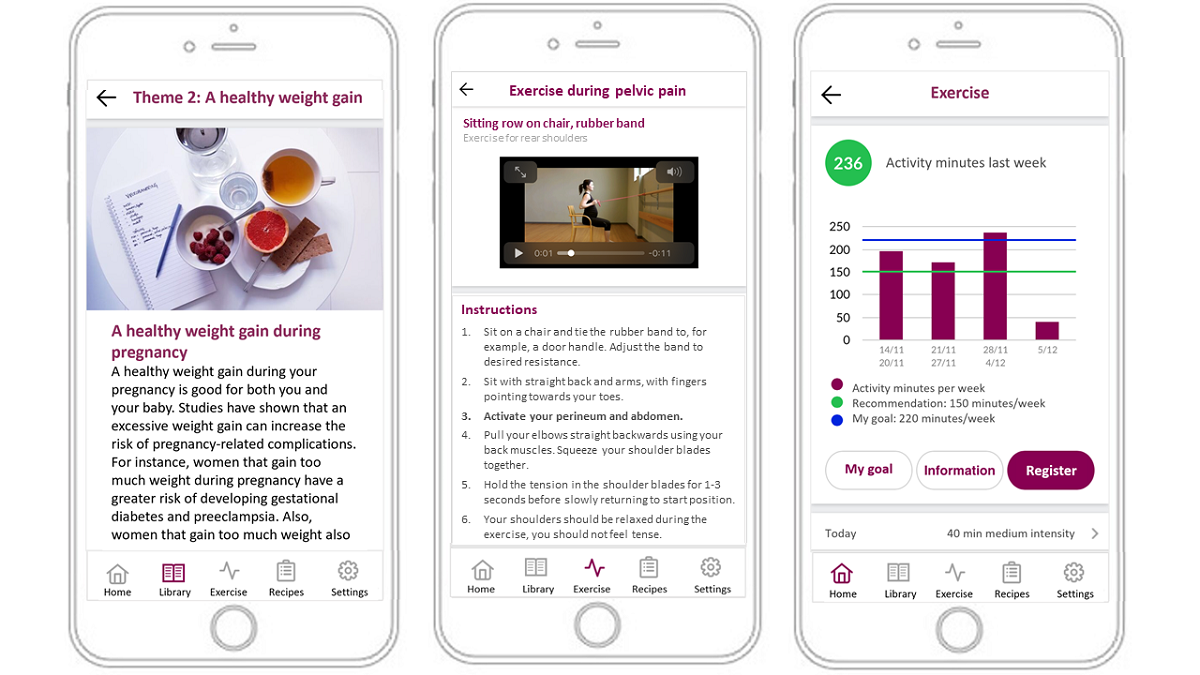
Illustration of 3 app features: information on why a healthy gestational weight gain is important (theme 2), the exercise feature with videos, and the physical activity registration with a goal-setting function and visual feedback in relation to recommendations.

The HealthyMoms trial (ClinicalTrials.gov NCT03298555) [17] was a 2-arm parallel randomized controlled trial that investigated the effects of a 6-month mHealth intervention (the HealthyMoms app) on GWG, diet, and physical activity. The study was initiated in October 2017, baseline measures (in gestational week 14) were completed in March 2020 (n=305), and follow-up measures (in gestational week 37) were completed in September 2020. After completion of the baseline measurements, participants were randomized (in a 1:1 ratio) to either the control or intervention group. The control group received standard maternity care and the intervention group received the HealthyMoms app (in addition to standard maternity care) for 6 months. The app was developed by researchers with expertise in nutrition, physical activity, pregnancy, and behavior change, and was grounded in social cognitive theory [19] and applied behavior change techniques [20]. HealthyMoms was built around 12 themes and included information and practical tips on diet, physical activity, and weight gain during pregnancy, with a new theme being introduced every other week. The app also included 3 registration features for diet, physical activity, and weight gain. In short, participants were encouraged to register their diet, physical activity, and weight weekly. Registration of diet was based on 5 questions regarding intakes of certain food groups (ie, fruit and vegetables, candy, snacks, sodas, and ice cream and pastries). For physical activity, participants imputed activity minutes as well as intensity of the exercise continuously during the week, and the registrations were summed up for that week. Following registration, participants received feedback based on current national guidelines for diet [21] and physical activity during pregnancy [22]. In the weight registration graph, participants could also view their weight gain compared with their recommended weight gain based on their prepregnancy BMI [5]. Furthermore, the app included an exercise feature (eg, videos and exercise programs), a recipe feature (eg, weekly menus and recipes), and a pregnancy calendar with weekly updates. Figure 1 illustrates 3 of the features (translated screenshots from Swedish to English) in the app. Participants also received push notifications 4 times per week (consisting of tips, encouragement, behavior change strategies, and reminders) and feedback from the registration of diet and physical activity based on national recommendations [21,22]. More detailed description of the rationale, development, and trial design and methods are available in the study protocol [17].

### Semistructured Interviews

Women in the intervention group were invited to participate in exit interviews at the follow-up measurement in gestational week 37 (August 2018 to February 2019). The Swedish female researcher (JS) working with the HealthyMoms trial was responsible for recruitment. A total of 20 participants were consecutively asked to participate and all agreed. One participant later declined to participate due to have given birth prior to the scheduled interview. During the final interviews, the research team experienced that no new information was gained and that further interviews were unlikely to provide additional information. We therefore considered that saturation was reached [23] and thus no more women were recruited.

Interviews took place in a separate room at Linköping University, Linköping, Sweden. Participants were interviewed individually and face to face in Swedish by JS. Interviews ranged between 14 and 49 minutes in duration (average time 29 minutes). A semistructured design was used; a set of main questions regarding the app’s layout and function and the participants’ usage, experiences, and satisfaction using the features in the app were asked, followed by questions tailored to individual responses. The interview guide (Multimedia Appendix 1) was developed by the research team, which has extensive knowledge in pregnancy, nutrition, physical activity, qualitative methodology, and mHealth. Prior to the interview, the participant was given a short introduction to the purpose of the interview and written informed consent was collected. All interviews were audiorecorded and fully transcribed by JS. Audio files were anonymized and kept stored unavailable to unauthorized people. The study was approved by the Regional Ethical Review Board in Linköping, Sweden, on April 24, 2017 (ref No. 2017/112-31), with an amendment on May 4, 2018 (ref No. 2018/262-32). The reporting of this study follows the Consolidated Criteria for Reporting Qualitative Research checklist (Multimedia Appendix 2) [24].

### Data Analysis

The semistructured interviews were analyzed using thematic analysis, as described by Braun and Clarke [25]. Data were analyzed using an inductive approach (ie, data-driven) at a semantic level. By using these approaches, there is a lower risk for the analysis to be affected by the preconceptions of the researchers [25]. Interviews were transcribed by JS (nutritionist, PhD student), who had previous experience conducting thematic analysis, and the transcribed texts were then actively read and reread several times by JS and EL (female medical student) to obtain a sense of the overall data. JS and EL separately coded interesting data (ie, text fragments from the transcribed texts) related to the aim of the study (ie, initial codes), and equal attention was given to each data item. The initial codes were then analyzed and sorted into groups to generate themes. Thereafter, any disagreements in the coding or grouping were discussed and resolved before preliminary themes were set. Themes were then reviewed, compared, and contrasted with support from A-KL, SR, ES, and ML, and the final result was derived from thorough discussions between the authors.

## Results

### Participants

The 19 interviewed women were representative of the whole intervention group (n=152) in terms of baseline characteristics such as average age (31.7, SD 4.4 years vs 31.4, SD 4.3 years), BMI (24.6, SD 3.4 kg/m^2^ vs 24.7, SD 4.3 kg/m^2^), educational attainment (13/19, 68% vs 115/152, 76% had a university degree), and parity (11/19, 58% vs 86/152, 57% were primiparous). Also, the 19 women participating in these interviews reported similar usage and satisfaction by means of a questionnaire after finalizing the intervention compared with women in the intervention group that completed the follow-up (n=134) [18]. For instance, they reported app usage comparable to the whole intervention group (15/19, 79% vs 111/134, 83% reported having used the app ≥1 time per week), and the majority reported that they agreed with the statement that they were satisfied with the app (18/19, 95% vs 122/134, 91%). Further, participants in the interview group and the whole intervention group agreed to a comparable extent with the statement that the app was a good support for healthy GWG (11/19, 58% vs 91/134, 68%, respectively).

### Themes From the Thematic Analysis

**Figure 2:**
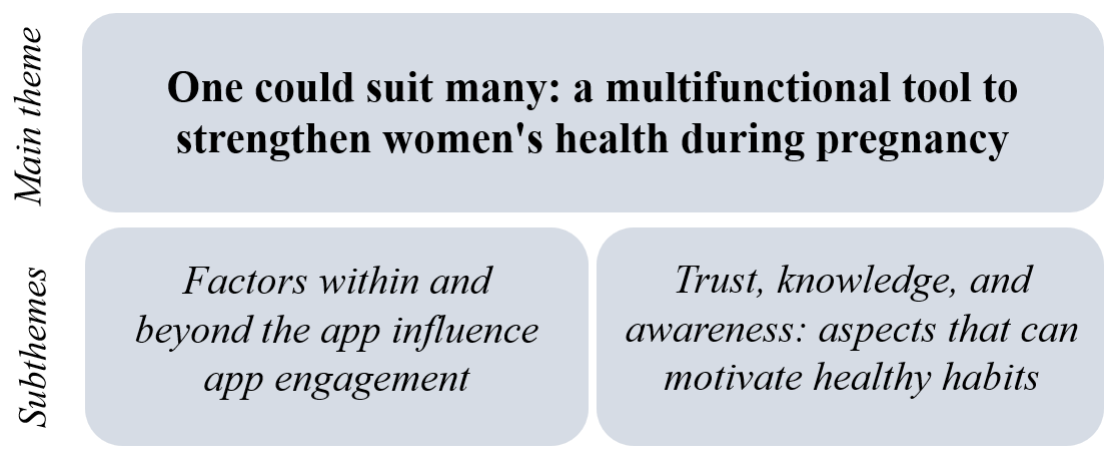
The themes identified in the thematic analysis.

One major theme and 2 subthemes were derived from the thematic analysis and are presented in Figure 2.

#### One Could Suit Many: A Multifunctional Tool to Strengthen Women’s Health During Pregnancy

The themes illustrated that a trustworthy and appreciated health and pregnancy app that was easy to use could inspire healthy habits during pregnancy. Overall, the app was appreciated but used in different ways. The first subtheme discussed factors within the app (eg, regular updates, feedback) and beyond the app (eg, motivation, interest, and the pregnancy itself) that influenced app engagement. The second subtheme reflected that trust, increased awareness, and knowledge were important and appreciated aspects in the app, as these motivated participants to improve or maintain healthy habits.

#### Factors Within and Beyond the App Influence App Engagement

Engagement with the app varied among participants, and several factors were described to influence app engagement. For instance, regular updates (with new app content and information) and push notifications were described as positive, as they sparked interest and increased usage. However, for some women, positive feedback on diet registrations actually decreased motivation if goals were repeatedly reached, which indicated no room for further improvement. Furthermore, when goals were not met, some women reported that feedback suggesting the need for improvement led to feelings of guilt and negative emotions, resulting in discontinued registration.

Too much time does not pass before you enter the app again, if it had I do not think I would have registered my own data as regularly, because otherwise I think you kind of would have forgotten that. So, I felt that I used the app more because of the push notifications.

Participant 5

Furthermore, the risk of becoming too fixated with weight and diet was mentioned as a reason for not engaging in these registration features. Compared with the other registration features, participants described being less motivated to use the diet registration because of difficulties remembering weekly food intakes and perceiving feedback as ambiguous. A more detailed diet registration was suggested as a potential improvement; however, it was also described that such registration could be too time-consuming and potentially put an unhealthy amount of focus on diet. Registration of physical activity was also described as confusing and choosing the appropriate intensity level was difficult. Thus, a suggested improvement was the option to choose the activity (eg, running, swimming, walking) instead of intensity level. Other suggested improvements were an inbuilt pedometer or the possibility of transferring data (manually or automatically) from other apps.

I did not quite understand the diet registration graph and because of that I could not tell if my diet improved or got worse. If it would have been easier to understand I think I probably would have been more motivated to continue to register my diet.

Participant 2

Several other factors beyond the app impacted usage. First, app engagement was described to be influenced by both lifestyle and prior knowledge as well as the experienced need of the features in the app (eg, need for motivation to exercise, to track GWG, or tips and information on how to eat healthy). For example, low or nonexistent usage of the diet registration could be explained by the lack of a need or motivation to make dietary changes and thus register diet. Furthermore, personal interests influenced app usage, especially the use of the recipe and exercise-related features.

The exercise functions were the best parts of the app, but that is also because I like to move and to be active, so that is probably the part that I have used the most.

Participant 15

Moreover, it was described that the usefulness of the app was greater when participants were motivated and experienced the need to change behavior. For instance, having gained too much weight during a previous pregnancy was described to motivate app usage. On the same note, parity was described to impact engagement and usefulness of the app, as women expecting their second child experienced less need of the pregnancy-related features compared with first-time mothers. Additionally, life situation (eg, number of children, workload) was mentioned to potentially impact app engagement, as it determined the available time to spend on an app. Nevertheless, both primiparous and multiparous women were satisfied with the app and found it to be useful.

I have not had the time to be active in any type of app or anything like that so I think that it is more those things [work, other children] that have made me feel that I have not had the time as I did during my first pregnancy when I literally read everything, so it has been a bit different this time.

Participant 14

Participants also reported that their engagement with the app changed during the course of their pregnancy. Curiosity about the app and the need of pregnancy-related information were higher in early pregnancy, but as women established new, healthier habits and perceived an increased sense of security as pregnancy progressed, app usage declined in late pregnancy. App engagement was sometimes negatively influenced by pregnancy complications (eg, nausea or pelvic pain), which could inhibit the ability and motivation to maintain a healthy lifestyle and consequently use the self-monitoring features in the app. Finally, initial higher app usage was explained mainly by participants having more energy and motivation in the beginning and middle of the pregnancy. In contrast, one participant described that the pregnancy initially felt more surreal, and as pregnancy progressed, engaging in healthy habits felt more important, and consequently app usage increased.

I was more curious of the app in the beginning, more curious of all the content, and then as time passed by I sort of only used the functions that I liked the most.

Participant 6

I was more committed [to the app] towards the end because then I kind of felt that it mattered more since I started to feel more pregnant.

Participant 5

#### Trust, Knowledge, and Awareness: Aspects That Can Motivate Healthy Habits

Participants stated that they appreciated the app because it had an appealing layout, was easy to use, had no technical shortcomings, and was perceived as a trustworthy source of information, as it was developed by experts. Furthermore, one participant described that the credibility of the app was strengthened by the fact that it was noncommercial. Another participant appreciated that the app content was in line with Swedish maternity care, which made the app more relevant and useful compared with other foreign pregnancy apps.

I’ve trusted this app considering the people behind it. If I am to be source-critical, I’ve found it very nice to have got it [my preconceptions] confirmed and I feel that I can trust that what I’m reading in the app is true.

Participant 9

Moreover, the app provided necessary information (eg, in the pregnancy calendar and themes). Thus, participants did not have to look for information elsewhere, which was considered valuable. The multifunctionality of the app, inclusion of registration features, and focus on both general and pregnancy-related health were appreciated and described as rare in other pregnancy apps. The wide range of features were also described to increase its usefulness, and thus the app could benefit many different women. Participants stated that HealthyMoms confirmed and increased their knowledge about healthy weight gain, diet, and physical activity during pregnancy, and increased awareness of how lifestyle could impact both maternal and fetal health was described as motivating. The registration features enabled self-evaluation and were considered a good support for changing or maintaining habits as well as supporting a healthy GWG. Participants reported that app usage had a positive impact on their diet and physical activity regardless of their habits prior to receiving the app.

I’ve become more motivated to eat well and exercise for my own health and because it [a healthy lifestyle] facilitates my pregnancy and also that it is good for the baby.

Participants 19

Additionally, information on the importance of healthy GWG was appreciated, and one participant described that it is not usually discussed at midwife appointments. However, the recommended GWG (based on the self-monitoring of weight in the app) was described as stricter than information received from the midwife, which brought up the importance of consistent information. Additionally, exceeding the GWG recommendations could cause feelings of anxiety, discouragement, and frustration.

I thought that [the recommended weight gain in the graph] was good because I do not feel that you get that information elsewhere, on how much weight you should gain, or that is something that is not spoken of at the midwife visits, well you talk about weight but not so much from that perspective.

Participant 12

Overall, self-monitoring was seen as motivating, as it increased awareness of GWG and dietary and exercise habits. On the same note, push notifications reminded participants of the importance of a healthy lifestyle, which was motivating; however, these could also be perceived as annoying when received at an inconvenient time (eg, receiving a message on the importance of a healthy diet right after having eaten something unhealthy). Moreover, self-monitoring and goal setting made the app feel personal, which was considered important. Participants described, however, that the app could be improved by adding additional personalized features, such as a calendar (to schedule, for example, exercise or midwife visits), tailored push notifications (eg, feedback related to registration), and challenges (eg, minor assignments such as going for a walk or having a piece of fruit).

Moreover, additional functions and information that were described as desirable included a larger focus on mental health, a sharing and network function, and more inclusion of the partner. Furthermore, to prevent the loss of healthy habits in early pregnancy, participants wished for earlier access to the app. A prolonged version covering the postpartum period to support a healthy lifestyle and the inclusion of baby-related advice were described as additional improvements.

I think it is important that it [the app] continues to be personal, that you feel that it is adapted to my premises and my starting weight, my BMI, and that you get to self-register as an individual and get data from that. I think that is the most important feature and what also perhaps separates this app from all other apps.

Participant 17

## Discussion

### Principal Results

Overall, this study demonstrated various levels of engagement and overall high satisfaction with the HealthyMoms app, and women perceived that it inspired healthy habits during pregnancy. Engagement with HealthyMoms was influenced by the app itself (eg, regular updates, feedback, and push notifications) and factors beyond the app (eg, interest, need, the pregnancy, and time constraints). Important and appreciated aspects of the app were trustworthiness, information, and features that increased awareness, knowledge, and motivation.

### Comparison With Prior Work

To the best of our knowledge, this is the first study to explore participants’ engagement and satisfaction related to long-term usage (ie, 6 months) of an mHealth app intended to support healthy weight gain and lifestyle during pregnancy. This study identified several factors both within and beyond the app that influenced engagement with the HealthyMoms app. Regular updates, push notifications, and feedback from the self-monitoring features sparked interest and positively influenced app engagement. Similarly, a study in a nonpregnant population found reminders to be positive; however, push notifications need to be carefully constructed in terms of timing and frequency, as they otherwise might be ignored [26]. This is similar to our study, where some participants described push notifications to be annoying when received at a bad time.

Although factors within the app influenced usage, factors beyond the app seemed to influence usage to a greater extent. Similar to a previous study exploring the acceptability of a multicomponent intervention in 9 Australian pregnant women [27], we also found that life situation and available time limited app engagement. Pregnancy itself was also described to guide app engagement; usage of the HealthyMoms app tended to peak in early pregnancy and then declined, which was explained by higher curiosity in the beginning. The same usage pattern has been seen in a previous study in Chinese pregnant women investigating the use of smartphone apps in general [28]. However, we also found that increased usage could occur during the final period due to feeling more pregnant and thus being more motivated to engage in healthy habits. Similar to previous studies investigating app usage in nonpregnant populations [26,29], app engagement was further described to be influenced by motivation, and usage could decrease with time due to the establishment of new habits. The usage of the HealthyMoms app also depended on pregnancy-related complications. These findings are similar to previous results by Willcox et al [27], in which participants described pregnancy-related physical ailments, such as back pain and morning sickness, as barriers to maintaining a healthy lifestyle. Consideration of these factors could be important for the development of future health and pregnancy apps.

Our findings show that the HealthyMoms app was perceived as trustworthy and a reliable source of information, as it contained evidence-based information and was developed by experts. This finding is supported by previous data on pregnant women’s app usage in general that have shown that unreliable or uncertain information can cause feelings of anxiety [28,30]. Furthermore, the importance of engagement from health care professionals and institutions in the development of this type of app have previously been emphasized [30,31]. Both users and health care professionals have been shown to prefer pregnancy apps that contain information related to their local health care context [30]. Likewise, participants in our study appreciated that the app was context specific and relevant to Swedish maternity care.

In line with previous studies [28,30-32], women in this study valued the HealthyMoms app for the wide range of features and its focus on both pregnancy and health. The opportunity to self-monitor was appreciated, and it increased awareness of dietary and exercise habits as well as GWG, which motivated a higher usage of the app and aided in establishing healthier habits. This is also in line with previous findings, which have reported that self-monitoring of weight during pregnancy could be perceived as helpful and motivating to stay within the recommendations for GWG [16]. However, the feature for weight registration could also be perceived as stressful and cause anxiety when exceeding the recommendations. In this study, participants also expressed that GWG in relation to the recommended weight gain is not usually spoken of in maternity care. Indeed, both midwives and pregnant women themselves have expressed challenges and stigma related to discussing GWG in maternity care [33-35]. Since GWG is important to address, an app with a carefully designed weight registration can fill an important gap.

Overall, participants were satisfied with the HealthyMoms app. However, improvements for future versions were identified. One function described as desirable was a sharing and network feature, which previously has been reported as an appreciated function by pregnant women [16,30,31]. Although the personalized aspects of the app (ie, the self-monitoring features) were highly appreciated, making goals and feedback more individualized was a suggested improvement. Likewise, individualization has been found to be important in previous studies in both pregnant [16] and nonpregnant populations [26]. Similar to a pilot study in Canadian women that evaluated an app (SmartMoms Canada) after a test period of 2 to 4 weeks [16], the participants in this study wished for a larger focus on mental health. This could be important to consider in future versions of the HealthyMoms app or other apps targeting pregnant women. Participants in our study wished for earlier access to the app in order to prevent the loss of healthy habits. They also expressed a desire for a continued version of the app after the baby’s arrival in order to support a healthy lifestyle after birth and for the app to include baby-related advice, which has been indicated as a requested feature in other populations of pregnant women [30,33]. Potentially, future app versions should be more comprehensive and span from early pregnancy into infancy.

In this study, the diet registration was perceived as difficult to use and should be improved in the future. A more detailed registration was suggested, but that could potentially lead to an unhealthy preoccupation with food. Moreover, our results suggest that self-monitoring of physical activity should include more detailed registrations, such as types of activity. Furthermore, an inbuilt pedometer or the opportunity to transfer data from other apps was expressed as a desirable addition. In comparison, Halili et al [16] combined an app (SmartMoms Canada) with a fitness tracker, which was appreciated by the pregnant women after a short test period. Ease of use and simplicity have also been found to be important in overcoming barriers (eg, lack of time) to app usage in nonpregnant populations [26]. Taken together, these suggested improvements could be important to consider for future version of the HealthyMoms app as well as future pregnancy and health apps.

### Strengths and Limitations

In this study, we have presented a qualitative evaluation of the engagement and satisfaction with HealthyMoms. This type of evaluation is important to guide future interventions, the development of other pregnancy and health apps, and future versions of HealthyMoms. Qualitative data also provide a more detailed evaluation of how women engaged with the program compared with quantitative measures only.

A strength of this study is the inclusion of 2 people (JS and EL) to undertake the thematic analysis, which allowed for constant validation during the process. The analysis was further strengthened by having 2 perspectives on the data, one from the person who conducted the interviews (JS) and one from someone who did not meet the participating women (EL); thus, comprehension of the data was discussed and validated with a low risk of biased interpretations.

This study was limited by the fact that only 19 out of the 134 participants in the intervention group participated in the interviews. However, importantly, the sample size was sufficient to obtain a broad and rich variety of experiences, the interviews demonstrated various engagement with the app as well as varying attitudes, and enough data were collected to achieve perceived saturation [23]. Additionally, the participants were representative of the intervention group. The participants had a higher education level compared with the general population, but this was similar to the education level in the entire group.

### Implications and Clinical Relevance

The HealthyMoms app was well received by participants and the multifunctionality of the app, including the self-monitoring features, were appreciated. Our results also provide valuable information that can be used to further iterate and improve the HealthyMoms app as well as guide the development of future pregnancy apps intended for long-term usage. Such minor improvements include more personalized features, a larger focus on mental health, and an inbuilt pedometer. Given that midwives may have little time to fully address and support healthy GWG in maternity care and due to the sensitive nature of weight gain, this app has the potential to fill an important gap. Considering that reducing the burden of excessive GWG has significant and important health implications for both mother and child [1,4], access to low-cost and scalable tools to promote healthy GWG is essential. The usage of pregnancy apps among women in developed countries is extensive [28,31,36], and a health and pregnancy app could therefore potentially reach many women.

### Conclusion

This study contributes novel information about pregnant women’s experiences using a health and pregnancy app intended to support a healthy GWG throughout pregnancy. The results showed that the HealthyMoms app was appreciated and is likely to benefit pregnant women. Several factors with both positive (eg, push notifications, regular updates, interest) and negative (eg, time constraints, pregnancy-related complications) impact on app engagement were observed. Trust, knowledge, and awareness were described as important aspects to motivate healthy habits. Taken together, our results showed that the app was considered a valuable support and could aid in establishing and maintaining healthy habits during pregnancy, which is important to prevent excessive GWG. Finally, our findings provide valuable information for the development of future pregnancy apps intended to support a healthy lifestyle.

## Appendix

Multimedia Appendix 1 The interview guide.

Multimedia Appendix 2 The Consolidated Criteria for Reporting Qualitative Research checklist.

## Abbreviations

GWG: gestational weight gain
mHealth: mobile health
