# Supplementary material for: Participants’ Engagement and Satisfaction With a Smartphone App Intended to Support Healthy Weight Gain, Diet, and Physical Activity During Pregnancy: Qualitative Study Within the HealthyMoms Trial
Source: JMIR Mhealth Uhealth. 2021 Mar 5;9(3):e26159. doi: 10.2196/26159 (PMC7980113; doi:10.2196/26159)
Supplement: Multimedia Appendix 1 [file mhealth_v9i3e26159_app1.docx]

**Multimedia Appendix 1.** The interview guide.

**Interview questions to the participants in the HealthyMoms intervention group**

You have now used the HealthyMoms app for 6 months and had access to the different features in the app. You have received messages, had the opportunity to set goals, view statistics on your weight gain, diet and exercise habits and received feedback. We are curious to know how you have experienced these features. Therefore, in today’s interview I will ask questions about this. Do you have any questions before we begin? Do you consent to participate in the interview and that it is being recorded (audio)?

1. If you think about the layout (the appearance), function and usability of the app, what grade would you give the app on a scale of 1 to 5? Feel free to think out loud and explain how you decided on that grade.
2. Alternative follow-up questions: How come you gave the app this grade? What would have had to be different in order for you to give the app a higher grade? What is it that does not make you give the app a lower grade?
3. Were the instructions on how to start using the app sufficient? If the answer is no, what information was missing?
4. What features in the app did you like the most and which did you find to be most useful?
5. What was good about these features?
6. Which features would you like to change? In which way?
7. In what way has the app influenced you?
8. The messages that you received from the app included: tips, reminders, information and strategies on diet, physical activity, a healthy gestational weight gain and how to change a habit. What messages do you remember and in what way were they meaningful to you?
9. Generally, what did you think of the content of the messages? In what way did the messages influence you? Follow-up question: What type of messages do you think would have influenced your diet- and exercise habits more?
10. Were there any types of messages that you missed? If so, what type?
11. What did you think of the number of messages? Would you have wanted to receive more/less messages?
12. Approximately how many of the messages did you read?
13. Was there a difference in how many messages you read in the beginning and in the end of the intervention? If yes, why do you think that is?
14. You could also set an exercise goal in the app (minutes per week). What did you think of this feature?
15. You had the possibility to register physical activity, diet, and weight gain in the app. Did you use these registration features? If yes, which ones? If no, why not? Is there anything that would have made you use them more?
16. In the app, you could view your exercise and dietary habits as well as weight gain over time (illustrated graphically) What did you think of these features? Were they easy or difficult to understand?
17. How did the feedback from the registration of diet and exercise affect you?
18. How did you like the weight registration feature? What did you think about the green field visible in the graph (the recommended weight gain)?
19. Has your usage of the app changed or looked different during the intervention period?
20. Would you have wanted to use the app more than you did? If yes, what would have had to have been different in order for you to have used the app more?
21. Have you used other health- and pregnancy apps during your pregnancy? If yes, which ones? If you would compare these apps with the HealthyMoms app – how would you say that they differ? What is better/inferior with the HealthyMoms app?
22. Do you experience that the usage of other apps (e.g., Facebook, Instagram, Snapchat, WhatsApp, etc.) affected how much you used the HealthyMoms app?
23. Would you recommend the app to someone close to you? Supplementary question: What is it that makes you recommend it? Alternative: What would have had to be different for you to recommend it?
24. In your opinion, what would a perfect health- and pregnancy app look like? What do you think are the three most important features in that kind of app?

***Thank you for participating in this interview!***
